# Perovskite-based electrochemiluminescence analysis of H_2_O_2_†

**DOI:** 10.1039/d4ra03652b

**Published:** 2024-06-19

**Authors:** Ziyi Jia, Hui Zhang, Yuxin Chen, Yuan Fang, Junnan Zhang, Shanwen Hu

**Affiliations:** a Department of Health Inspection and Quarantine, School of Public Health, Fujian Medical University Fuzhou Fujian 350122 P. R. China shanwenhu@fjmu.edu.cn

## Abstract

The detection of hydrogen peroxide (H_2_O_2_) represents an extensive requirement across various domains, including food, environmental, and medical fields. This study introduces a highly sensitive technique for the quantification of H_2_O_2_, integrating the electrochemiluminescence properties of perovskite with bio-catalyzed precipitation. A water-soluble perovskite-based electrochemiluminescence (ECL) biosensing interface was constructed, wherein H_2_O_2_ catalyzes a precipitation reaction that leads to the formation of an insoluble precipitate on the electrode surface. This occurrence effectively quenches the electrochemiluminescence signal of the perovskite, thus facilitating the quantitative detection of H_2_O_2_. The modified perovskite demonstrated excellent ECL performance, offering a stable signal source, while the bio-catalyzed precipitation reaction significantly amplified the quenching effect, thereby enhancing detection sensitivity. This strategy exhibits excellent stability and sensitivity, presenting a promising method for the detection of hydrogen peroxide, which holds great potential for applications in various fields.

## Abstract 2

The detection of hydrogen peroxide (H_2_O_2_) represents an extensive requirement across various domains, including food, environmental, and medical fields.

## Graphical abstract

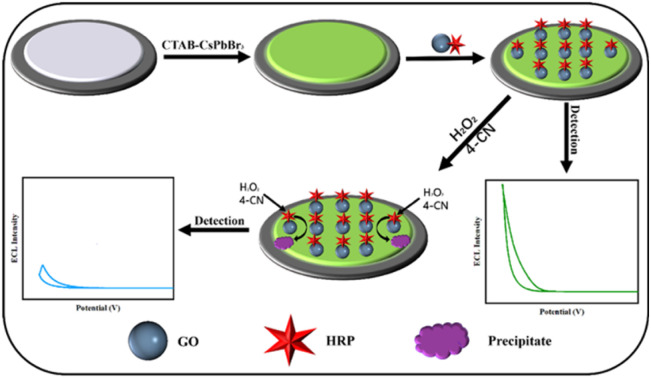

## 1. Introduction

Hydrogen peroxide (H_2_O_2_), a widely employed oxidizing and disinfecting agent, is crucial to the pharmaceutical industry, healthcare, and food manufacturing. Additionally, it serves as a byproduct in various enzymatically catalyzed metabolic processes. Precise measurement of H_2_O_2_ levels is vital in diagnosing infections, monitoring wound healing, or evaluating the efficacy of disinfection procedures. Moreover, in the food and pharmaceutical industries, the detection of H_2_O_2_ can reveal the efficiency of cleaning and sterilization processes, ensuring product safety and quality. Within environmental analysis, measuring H_2_O_2_ aids in water quality assessment, pollution source identification, and monitoring oxidative stress in aquatic ecosystems. Notably, as a by-product of human cellular metabolism, endogenous H_2_O_2_ is a natural cellular process product,^1^ which can become harmful if its levels exceed a certain threshold. Excessive levels of H_2_O_2_ can lead to oxidative stress, causing cellular damage and potentially contributing to the development of various diseases. Therefore, accurate monitoring of H_2_O_2_ is significant for diverse biological processes.

Approaches for detecting hydrogen peroxide primarily include colorimetry, fluorescence, and ECL. Anteneh F. Baye and colleagues have employed nitrogenous environments for the carbothermal reduction of ZnFe double hydroxide and glucose-derived carbon to fabricate Fe_3_O_4_–Fe^0^/Fe_3_C nanozymes, constructing a colorimetric sensor for H_2_O_2_ that offers high selectivity and ultra-sensitivity.^2^ Di Cheng and associates reported on CuCo_2_O_4_'s excellent electrocatalytic activity towards H_2_O_2_, establishing an electrochemical sensor based on CuCo_2_O_4_ that demonstrated a linear range of detection from 10.0 µM to 8.9 mM and a sensitivity of 94.1 µA mM^−1^ cm^−2^.^3^ Among these methods, colorimetry, with its low cost, convenience, and independence from large-scale instrumentation, is suited for the onsite instantaneous detection of hydrogen peroxide, despite its limited detection range and dissatisfactory detection limits. While fluorescence offers a lower detection limit, its results are susceptible to interference from intrinsic fluorescence backgrounds. ECL (electrochemiluminescence), combining advantageous properties such as good controllability, high electrochemical sensitivity, rapid response, and high specificity, alongside easy operation and ultra-high sensitivity, has been widely applied for H_2_O_2_ detection. In ECL detection, the luminescent material and the recognition reaction constitute two crucial factors determining sensitivity. Wang and coworkers' use of a graphene–cadmium sulfide (G–CdS) nanocomposite material reacting with H_2_O_2_ produced a strong and stable ECL, thereby enhancing the ECL intensity by approx. 4.3 times and significantly lowering the initiation potential, with excellent reproducibility and long-term stability.^4^ Wang *et al.* have developed an ECL biosensor for high-sensitivity detection of H_2_O_2_ in living cells. The biosensor is based on CdZnSeS quantum dots (QDs) and ordered mesoporous carbon (OMC). The study demonstrates the potential and reliability of the biosensor in diagnostic applications and physiological research.^5^ The team of Han, Jee Noon investigated the ECL of luminescent phenols on mesoporous platinum electrodes under neutral conditions. In the presence of hydrogen peroxide, the ECL reaction of luminescent phenols is more intense and exhibits a linear relationship with good reproducibility.^6^ Liu have developed a non-enzymatic ECL sensor based on single-stranded DNA (ssDNA)/g-C_3_N_4_ nanosheets. The sensor exhibits high peroxidase-like activity, which catalyses the ECL reaction of luminescent phenol–H_2_O_2_ on a glass carbon electrode after modification. This amplifies the luminescence signal and reduces the detection limit.^7^

Enhancing the sensitivity of H_2_O_2_ detection is paramount for early detection and accurate quantification, and numerous efforts have been devoted to this issue. One approach is to construct an enhanced signal related to the target molecules, such as using more efficient electrode materials in electrochemical sensors. Perovskite compounds have garnered increasing attention for their unique ECL performance. Specifically, halide perovskites, particularly all-inorganic cesium lead halide nanocrystals (CsPbX_3_, X = Cl, Br, I), have attracted widespread interest due to their adjustable band gaps, narrow emission bandwidths, excellent thermal stability, superior carrier transport properties, and high photoluminescence (PL) quantum yields. These exceptional physical and optical properties of perovskite nanomaterials, especially their ultrahigh PLQYs, render them potential novel ECL luminophores. The pioneering study utilizing perovskites for electrochemical detection of hydrogen peroxide was published by Shimizu in 1996, demonstrating that electrodes based on carbon-loaded La_0.6_Ca_0.4_Ni_0.7_Fe_0.3_O_3_ could produce the highest performance potentiometric H_2_O_2_ sensors.^8^ Research by He, J employed 800 nm PMMA microspheres to synthesize 3DOM-SmCoO_3_ perovskite oxide electrodes for H_2_O_2_ detection, not only overcame various drawbacks associated with enzyme-based and precious metal-based sensors but also achieved lower costs and higher speeds.^9^ Wang, XT, and colleagues utilized a sol–gel method to prepare two differently structured layered perovskite nanocrystals, modified on bare glassy carbon electrodes (GCE), assembling a highly efficient enzyme-free electrochemical sensor for H_2_O_2_, achieving trace detection through its high selectivity and stability.^10^ Wang, HY's team fabricated a non-enzymatic electrochemical sensor for H_2_O_2_ detection based on nickel-based rare-earth perovskite LaNiTiO_3_ nanoparticles, which exhibited good catalytic performance and detection advantages for H_2_O_2_ in a 0.1 mol L^−1^ NaOH solution.^11^ Different types of perovskite are developed to improve the electrochemistry properties for better analysis performance. Cao *et al.* have developed a self-enhanced LCPB-OAm ECL system with high-quality CsPbBr_3_ perovskite nanocrystals as novel ECL emitters, for the bioanalyses of hydrogen peroxide based on inhibition.^12^ Results of Wang *et al.* shed the light on the electrochemically switchable H_2_O_2_ ECL sensing application of the high-throughput microwave-synthesized IPQDs.^13^ Efforts have also been devoted to explore the electrochemical process between perovskite and H_2_O_2_. Chen studied the electrochemical redox and charge transfer natures of Rb_*x*_Cs_1−*x*_PbBr_3_ NCs in an ECL method, indicating the Rb_0.2_Cs_0.8_PbBr_3_ NCs have a higher ECL intensity than the CsPbBr_3_ NCs. The tunable ECL properties of the Rb_*x*_Cs_1−*x*_PbBr_3_ NCs are helpful to design novel ECL emitters in H_2_O_2_ ECL analysis.^14^ Qiu *et al.* unveiled the interfacial electrochemiluminescence behavior of lead halide perovskite nanocrystals and H_2_O_2_.^15^ Huang's team explored hydrogen peroxide involved anodic charge transfer and electrochemiluminescence of all-inorganic halide perovskite CsPbBr_3_ nanocrystals in an aqueous medium.^16^

Although there are already numerous methods available for constructing highly sensitive ECL systems using perovskite, there are still need to improve the sensitivity of detecting hydrogen peroxide. In this study, we comprehensively considered both the ECL system and the recognition process of target molecule. On the one hand, we synthesized CTAB-modified three-dimensional perovskites *via* a hot injection method, enhancing the water solubility of perovskites for more stable electrode modification, and on the other hand, we utilized hydrogen peroxide to induce a precipitating reaction with a high quenching efficiency, to construct a quantitative detection strategy for hydrogen peroxide.

## 2. Experiment

### 2.1 Perovskite synthesis

The synthesis of CTAB-PNC was carried out using a classic hot-injection method with slight modifications to the original protocol.^17,18^ Precursor synthesis: 0.22 g of cesium carbonate (Cs_2_CO_3_) was added to a single-neck flask containing 10 mL of octadecene (ODE) and 0.65 mL of oleic acid (OAc). The mixture was heated to 120 °C under a nitrogen atmosphere for 20 minutes, then the temperature was raised to 150 °C and maintained for another 20 minutes. After stopping the reaction, the precursor was kept sealed to minimize air contact and heated to complete dissolution before use. Synthesis of CsPbBr_3_ perovskite: 0.104 g of lead bromide (PbBr_2_) was added to a three-neck flask containing 10 mL of octadecene, 1 mL of oleic acid, and 1 mL of oleylamine mixture. The mixture was heated to 120 °C under vacuum and held for 1 hour, then the temperature was raised to 170 °C. After adding 0.8 mL of the precursor, the mixture was kept for 5 seconds then quickly cooled in an ice bath. The crude solution was centrifuged at 8000 rpm for 10 minutes to collect the precipitate, which was then dispersed in *n*-hexane.

### 2.2 GO–HRP composite synthesis

To maintain the activity of horseradish peroxidase, the pH of graphene was first adjusted to 7.4. 11 mg of EDC (1-(3-dimethylaminopropyl)-3-ethylcarbodiimide) and 15 mg of NHS (*N*-hydroxysuccinimide) were mixed with 1 mg mL^−1^ graphene solution (pH = 7.4) and stirred for 60 minutes. Then, 30 µL of 10 mg mL^−1^ HRP solution was added dropwise to the stirred graphene solution and stirred at a speed of 150 rpm for 12 hours. After the reaction was completed, it was stored at 4 °C for later use.

### 2.3 ECL sensing

#### 2.3.1 Preparation of electrodes

Glassy carbon electrodes were polished with 0.3 µm and 0.05 µm alumina slurries, respectively. The polished glassy carbon electrodes were then ultrasonically cleansed in 50% nitric acid solution, anhydrous ethanol, and pure water, then dried with nitrogen gas. A solution of 5 mM potassium ferricyanide in 0.1 M KCl was used as the electrolyte to scan the cyclic voltammogram (scan range from −0.2 V to 0.6 V at a scan rate of 50 mV s^−1^). The electrochemical impedance was scanned in the range from 0.1 Hz to 1000 kHz with an AC amplitude of 0.005. 2.5 µL of the perovskite dispersion was dropped onto the polished glassy carbon electrode and dried at room temperature to get the perovskite-modified electrode. A three-electrode system was used with the glassy carbon electrode as the working electrode, a platinum wire electrode as the counter electrode, and an Ag/AgCl electrode as the reference electrode.

#### 2.3.2 Construction of ECL biosensing interface

2.5 µL of the perovskite solution was dropped onto the electrode and air-dried overnight at room temperature before activating with NHS. The prepared GO–HRP composite was then dropped onto the activated electrode and reacted overnight at 4 °C. After the reaction was complete, the electrode was kept aside for further use. The perovskite electrode with HRP was immersed in 10× PBS solution containing 0.2 M potassium persulfate as the electrolyte to measure electrochemical luminescence, scanning the electrode potential from 0 to −2.45 V, while the photomultiplier tube (PMT) voltage remained at 380 V and the scan rate was 0.1 V s^−1^, producing an initial signal. A mixture of 1 mM 4-CN (4-chloro-1-naphthol) and different concentrations of H_2_O_2_ was prepared. 10 µL of the mixture was applied to the HRP-carrying perovskite electrode for a certain period to measure the electrochemical luminescence signal.

## 3. Results and discussions

### 3.1 Principle of the ECL analysis of H_2_O_2_

**Fig. 1:**
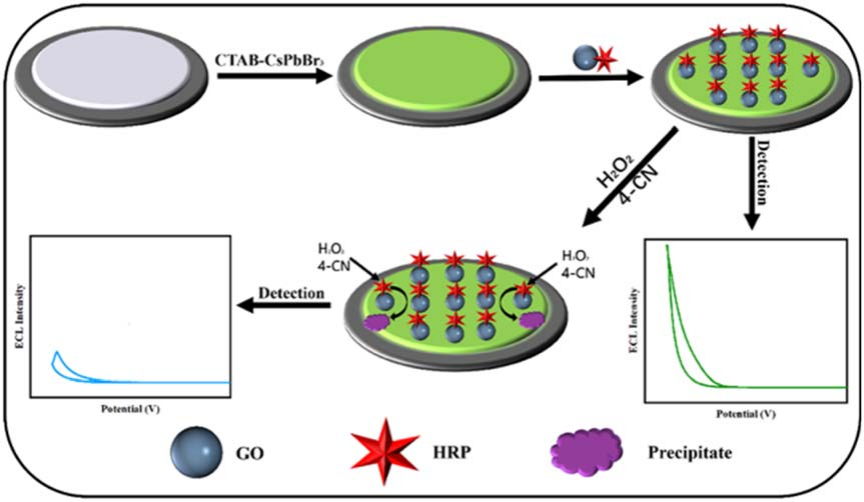
Illustration of ECL detection for hydrogen peroxide.

The principle of ECL detection in this study is depicted in Fig. 1. Initially, three-dimensional perovskite modified with cetyltrimethylammonium bromide (CTAB–CsPbBr_3_) was synthesized using a hot-injection method. The pristine CsPbBr_3_ suffers from poor photo-stability and water-stability due to surface defects. The introduction of CTAB passivates the surface defects of CsPbBr_3_ by forming a stable self-assembled monolayer on the perovskite surface,^19,20^ significantly enhancing its photo-stability and water-stability. The synthesized perovskite quantum dots are first drop-cast onto a glassy carbon electrode, which is crucial for generating the ECL signal. To immobilize horseradish peroxidase onto the electrode, a covalent complex of horseradish peroxidase and carboxylated graphene oxide (GO–HRP) was prepared in advance *via* the carbodiimide reaction. GO–HRP was then immobilized onto the activated electrode modified with CTAB–CsPbBr_3_. In the presence of hydrogen peroxide and 4-CN, the HRP on the electrode catalyzes the oxidation of 4-CN by hydrogen peroxide, generating insoluble and insulating benzo-4-chlorohexadienone products on the electrode surface. This results in a shading effect that influences the ECL intensity of the immobilized CTAB–CsPbBr_3_ on the electrode surface, causing a decrease in the ECL signal negatively related with H_2_O_2_ concentrations.

### 3.2 Characterization of the CTAB–CsPbBr_3_

**Fig. 2:**
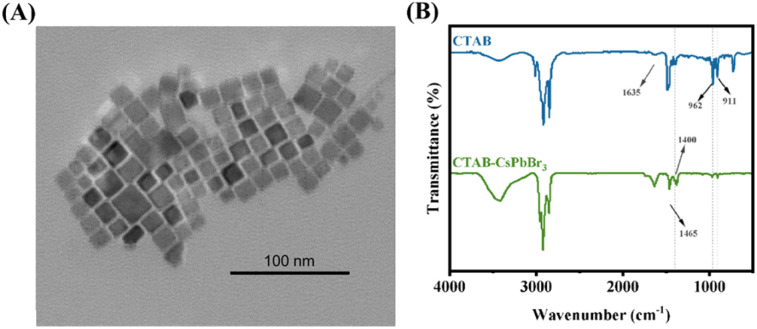
(A) The TEM image of CTAB–CsPbBr_3_; (B) the FTIR spectrometer image of CTAB (blue line) and CTAB–CsPbBr_3_ (green line).

Appropriate surface modification can improve the physicochemical properties of nanomaterials, thus enhancing the materials' testing performance.^21,22^ TAs shown in Fig. 2A, the crystal structure of CTAB–CsPbBr_3_ under the transmission electron microscope (TEM) exhibits regular rectangular shapes, with a size of approximately 15 nm. To verify the passivation effect of CTAB on the surface of CsPbBr_3_, Fourier Transform Infrared Spectroscopy was employed to characterize the surface ligands of the synthesized CTAB-modified perovskite.^20^Fig. 2B shows absorption bands at 1540 cm^−1^ and 1400 cm^−1^ for the CTAB–CsPbBr_3_ sample, owing to the asymmetric and symmetric stretching vibrations of the carboxylate groups, indicating the complexation of oleate anions on the surface of CsPbBr_3_ NCs.^23^ The spectrum of CTAB-modified CsPbBr_3_ NCs exhibited strong signals at 2850 cm^−1^ and 2920 cm^−1^, attributed to the symmetric and asymmetric stretching vibrations of CH_2_ and CH_3_, respectively, elucidating the interaction of oleic acid and oleylamine ligands with the perovskite crystals. Absorption peaks at 911 cm^−1^ and 962 cm^−1^ corresponding to the stretching vibrations of C–N^+^ are also visible, signifying typical absorption bands of CTAB. Notably, the spectrum of CTAB–CsPbBr_3_ features a distinct absorption peak at 1635 cm^−1^, corresponding to the asymmetric deformation of amino groups.

**Fig. 3:**
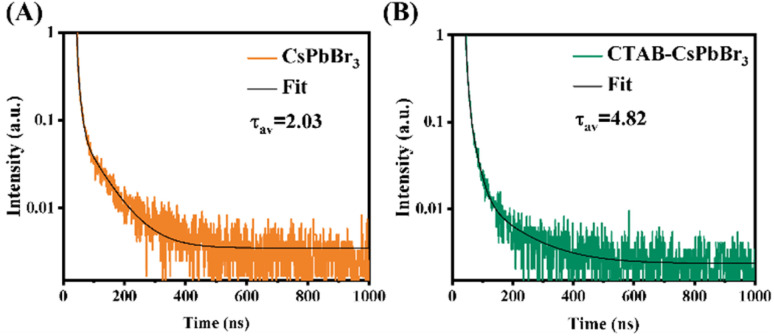
(A) Time-resolved PL decay of CsPbBr_3_; (B) time-resolved PL decay of CTAB–CsPbBr_3_.

To investigate the effect of CTAB on CsPbBr_3_, fluorescence lifetimes of both CTAB–CsPbBr_3_ and CsPbBr_3_ were tested and compared. The time-resolved PL decay curves in Fig. 3 were fitted with a tri-exponential decay function using a nonlinear fitting algorithm, which yielded good agreement with the experimental data (*R*^2^ all above 0.995). The average fluorescence lifetime was calculated using the following formula:
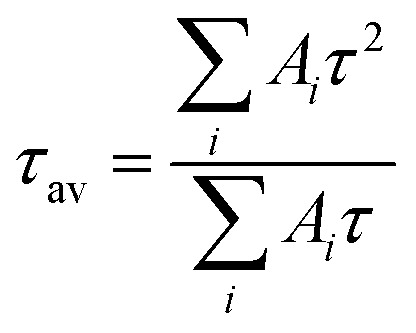


As shown in Fig. 3, the PL decay rate of the CTAB-modified perovskite was slower compared to CsPbBr_3_, extending the average fluorescence lifetime from 2.03 ns to 4.82 ns. This result demonstrates that CTAB passivation enhanced the stability of CsPbBr_3_. The three lifetimes, *τ*_1_, *τ*_2_, and *τ*_3_, obtained from the fitting curve using a tri-exponential decay function, represent the intrinsic exciton relaxation, the interaction between excitons and phonons, and the interaction between excitons and defects.^24^ In Table S1,† the *τ*_1_, *τ*_2_, and *τ*_3_ of CsPbBr_3_ were 2.03 ns, 8.96 ns, and 71.38 ns, respectively; whereas for CTAB–CsPbBr_3_, *τ*_1_, *τ*_2_, and *τ*_3_ were 4.80 ns, 19.87 ns, and 122.87 ns, respectively. Surface defects on the CsPbBr_3_ nanocrystal materials not only shorten the PL decay lifetime but also decrease the luminous efficiency of the nanocrystals.^25^ The surface modification of CTAB–CsPbBr_3_ exhibited an extended fluorescence lifetime, reducing non-radiative recombination and thereby enhancing the luminous efficiency of CsPbBr_3_.

### 3.3 Characterization of the electrode

The characterization was performed using differential pulse voltammetry (DPV) to measure the electrochemical signals of the perovskite-modified electrode with and without the presence of potassium persulfate. The DPV curves in Fig. S2† show the responses of the perovskite-modified electrode in potassium persulfate solution and in PBS solution without potassium persulfate. As demonstrated, a distinct cathodic peak appears for the perovskite-modified electrode in the presence of potassium persulfate solution; in contrast, no significant peak is observed in the absence. The DPV results confirm that the addition of the co-reactant potassium persulfate alters the electrochemical process of the perovskite-modified layer, which promotes electron transfer of modified electrode, thereby generating a significant peak.

**Fig. 4:**
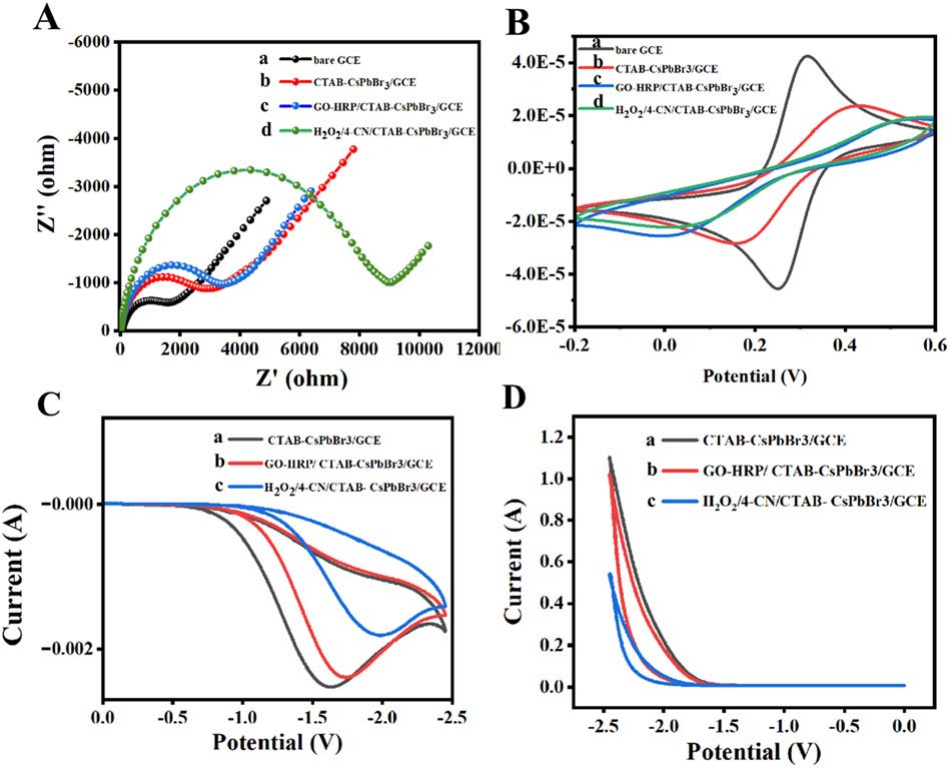
Electrical characterization of modified electrodes. (A) EIS spectra and (B) CV curves of various modified electrodes in 0.1 M KCl solution containing ferricyanide (a–d: bare GCE, CTAB–CsPbBr_3_/GCE, GO–HRP/CTAB–CsPbBr_3_/GCE, H_2_O_2_/4-CN/CTAB–CsPbBr_3_/GCE). (C) CV curves and (D) ECL spectra of CTAB–CsPbBr_3_/GCE (curve a), GO–HRP/CTAB–CsPbBr_3_/GCE (curve b), and GO–HRP/CTAB–CsPbBr_3_/GCE (curve c) after incubation with 4-CN and H_2_O_2_ in 0.2 M potassium persulfate solution.

Recording electrochemical impedance spectroscopy (EIS) graphs of various modified electrodes is an effective method to study electron transfer efficiency. To demonstrate the occurrence of precipitation reactions on the electrode surfaces, EIS measurements were conducted for differently modified electrodes in a potassium ferricyanide solution containing 0.1 M KCl. Within the impedance spectra, the high-frequency region correlates with rapid charge transfer processes in the electrochemical system, whereas the low-frequency region pertains to slower charge transfer processes, with the semicircular shape at low frequencies representing interface phenomena that restrict charge transfer.^26^ As shown in Fig. 4A, the impedance spectrum exhibits an increasing semicircle with each successive layer of modification on the electrode surface, indicating the successful assembly of horseradish peroxidase (HRP) and the formation of insoluble precipitate benzo-4-chlorohexadienone.^27^ The increase in resistance is attributed to the poor conductivity of the enzyme and the inhibition of electron transfer by the gradually increasing resistive effects of the insoluble precipitate layer.^28,29^

Cyclic voltammetry (CV) is an important method for validating electrochemiluminescence sensors. Experiments were performed in a potassium ferricyanide solution with 0.1 M KCl, and the CV test results are presented as indicated in Fig. 4B. Compared to the bare electrode (curve a), the redox peak current of the perovskite-modified electrode significantly decreased, confirming the successful modification with ECL material. Subsequently, the current response decreased after immobilizing GO–HRP on the electrode surface, due to the insulating properties of HRP affecting electron transfer at the electrode surface. Finally, in the presence of both hydrogen peroxide and 4-CN, HRP on the electrode surface catalyzed the oxidation of 4-CN by hydrogen peroxide to produce a precipitate, resulting in a continuous decrease in peak current response. Both electrochemical impedance and cyclic voltammetry outcomes confirm the occurrence of the precipitation reaction on the electrode surface.

To assess the ECL performance of the sensor and verify the impact of precipitation reaction on the perovskite signal, experiments were conducted using differently modified electrodes in a 0.2 M potassium persulfate solution. Initially, CV curves were collected (Fig. 4C), where the electrode solely modified with CTAB–CsPbBr_3_ exhibited a strong cathodic peak in the solution containing the co-reactant due to the facilitated charge transfer process. Subsequently, upon immobilizing GO–HRP on the electrode, a layer was formed obstructing the electrode surface, which restricted the charge transfer rate, resulting in a slight decrease in the CV curve peak. Finally, when the GO–HRP/CTAB–CsPbBr_3_/GCE was reacted with H_2_O_2_ in the presence of 4-CN, a significant decrease in the CV curve peak was observed, indicating the oxidation of 4-CN at the electrode surface forming an insoluble precipitate, thereby inhibiting electron transfer.^30^

ECL curves of different electrodes in PBS solution containing potassium persulfate were collected. As shown in Fig. 4D, the ECL signal of CTAB–CsPbBr_3_/GCE was remarkably significant, demonstrating the strong ECL property of perovskite in the presence of the co-reactant. With the immobilization of GO–HRP, the ECL signal slightly decreased due to the increased resistance at the electrode surface, which slowed down the electron transfer. When GO–HRP/CTAB–CsPbBr_3_/GCE reacted with H_2_O_2_ in the presence of 4-CN, the formation of an insoluble precipitate inhibited charge transfer, leading to a rapid decrease in ECL signal.^31^ These results collectively prove that the precipitation reaction on the electrode was successfully completed as expected, ultimately leading to a notable negative correlation signal.

### 3.4 Condition optimization

Fig. S3† respectively illustrates the effects of scan rate and scan potential range on the electrochemiluminescence intensity of the perovskite-modified electrode. As shown in Fig. S3A,† at very low scan rates, due to the slower rate of charge transfer at the electrode surface, fewer potassium persulfate molecules participate in the reaction, leading to a lower number of electrons and holes injected into CsPbBr_3_, thus resulting in low and unstable electrochemiluminescence efficiency. With the increase in scan rate, more potassium persulfate molecules are involved in the reaction, enhancing the efficiency of the charge injection process, thereby yielding stable and higher electrochemiluminescence signals. However, when the scan rate exceeds 0.1 V s^−1^, the rate of the electrochemical reaction may surpass the recombination rate of electrons and holes on the perovskite surface, leading to ineffective recombination of electrons and holes within the crystal and consequently diminishing the electrochemiluminescence efficiency and signal strength.

As depicted in Fig. S3B,† when the scan potential range is too narrow, the speed of the electrochemical reaction is low, thus limiting the quantity of electrons and holes injected into CsPbBr_3_, resulting in a weak ECL signal due to the limited formation of excitons in CsPbBr_3_ NC*. With an increase in the scan potential range, the rate of charge transfer accelerates, enhancing the amount of electrons and holes injected into CsPbBr_3_, and subsequently increasing the formation of CsPbBr_3_ NC* products, leading to a gradual increase in the electrochemiluminescence signal. On the other hand, an excessively wide scan potential range may lead to unstable intermediates or side reactions, adversely affecting the electrochemiluminescence signal. Following optimization of the scan rate and scan voltage, the optimal scan rate was determined to be 0.1 V s^−1^ with a scan voltage range from 0 to −2.45 V.

In order to achieve optimal analytical performance, the study conducted an optimization of the concentrations of HRP and 4-CN, as well as the reaction time involved. HRP plays a pivotal role in the precipitation reaction that occurs on the electrode surface. During the synthesis of GO–HRP, products synthesized with various concentrations of HRP were used. As demonstrated in Fig. S3C,† the GO–HRP synthesized using an ultimate concentration of 300 µg mL^−1^ of HRP, when immobilized on the electrode, exhibited the most effective catalysis of the H_2_O_2_-mediated oxidation of 4-CN, resulting in the greatest decrease in ECL signal and the highest quenching efficiency.

With the GO–HRP synthesized using the optimum HRP concentration immobilized on the electrode, the concentration of 4-CN was varied. Fig. S3D† indicates that at a 4-CN concentration of 1 mM, the most significant decrease in ECL signal was observed, suggesting the most pronounced effect of the precipitation reaction on the ECL signal.

**Fig. 5:**
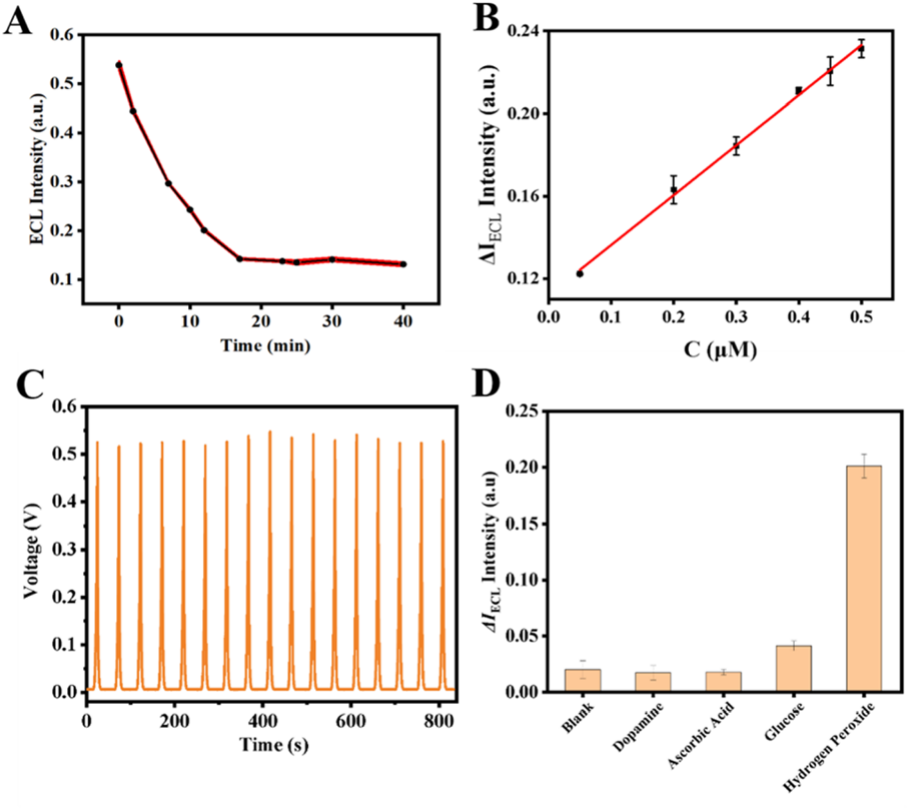
(A) Optimization of reaction time for detecting H_2_O_2_ by electrochemical luminescence method; (B) standard curves obtained by the detection of different concentrations of H_2_O_2_; (C) ECL-time response curve of H_2_O_2_ sensor after 17 consecutive scanning cycles; (D) ECL sensor specificity and selectivity (where H_2_O_2_ concentration was 0.35 µM; the concentration of other interfering substances was 3.5 µM.).

Finally, at room temperature, H_2_O_2_ and 4-CN were added to the electrolyte solution, and the variations in the ECL signal of the GO–HRP/CTAB–CsPbBr_3_/GCE were monitored over a reaction time of 0–40 minutes. As indicated by Fig. 5 A, the ECL signal showed a pronounced decreasing trend over the time period from 0 to 17 minutes; after 17 minutes, the ECL signal gradually stabilized.

### 3.5 ECL detection performance

Under the optimized conditions elucidated above, the sensing performance of the ECL sensor was evaluated. Initially, various concentrations of H_2_O_2_ were detected. As shown in Fig. 5B, an increase in the concentration of H_2_O_2_ led to an increase in the precipitate reaction products on the electrode, which in turn enhanced the quenching effect on the perovskite ECL, resulting in a more pronounced decrease in the ECL signal. Within the range of 0.05 µM to 0.5 µM, a favorable linear relationship between ΔECL intensity and H_2_O_2_ concentration was observed. By calculating the difference in ECL signal intensity before and after the reaction with H_2_O_2_, a linear regression equation was constructed for Δ*I*_ECL_ against H_2_O_2_ concentration, with the equation being Δ*I*_ECL_ = 0.24213*C* + 0.11211 (*R*^2^ = 0.997). The detection limit (LOD) was determined to be 50 nM.

Additionally, assessments of stability and selectivity are indispensable. To evaluate the stability, the prepared electrode was continuously scanned for 17 cycles in a potassium persulfate solution. No significant variation was noted in the ECL sensor signal over 17 consecutive cycles, with the relative standard deviation (RSD) calculated at 1.64%, indicating good stability, as illustrated in Fig. 5C. And the long-time stability was demonstrated in Fig. S4.† For selectivity, potential interferents such as dopamine, ascorbic acid, and glucose were used. The concentration of H_2_O_2_ was set at 0.35 µM, whereas the concentrations of the other interfering substances were established at 3.5 µM each. As depicted in Fig. 5D, the ECL responses to the interfering substances showed no significant deviation from the ECL signal of the blank sample. However, upon the addition of H_2_O_2_ at a concentration tenfold lower than that of the interfering substances, the sensor still demonstrated a strong response, signifying outstanding selectivity for H_2_O_2_.

### 3.6 Sample validation of the approach

To further evaluate the capability of the ECL sensor in complex samples, the method was applied to the detection of H_2_O_2_ in milk samples under optimal conditions using the standard addition method. As shown in Table 1, the recovery rates ranged between 98% and 106%, with RSD values below 4%. These results indicate a potential application in food analysis.

**Table 1:** Sample detection results of H_2_O_2_

Sample	Spiked (µM)	Detected (µM)	Recovery (%)	RSD (*n* = 3, %)
1	0.07	0.069	98.5	1.7
2	0.16	0.170	106	3.9
3	0.36	0.369	103	2.6

## 4. Conclusions

In conclusion, perovskite-based ECL approach for the detection of hydrogen peroxide was proposed. A water-soluble perovskite was used to construct a sensitive ECL interface on electrode, and the H_2_O_2_-catalyzed precipitation reaction, resulting in the formation of an insoluble precipitate on the electrode surface and quenching the ECL signal, thereby enabling precise detection, offering a novel approach for the monitoring of hydrogen peroxide, which holds significant potential for applications in food safety, environmental monitoring, and other fields.

## Data availability

The data that support the findings of this study are available on request from the corresponding author.

## Author contributions

Ziyi Jia and Hui Zhang: proposing and conducting experiments, writing original draft. Yuxin Chen: investigation. Junnan Zhang and Yuan Fang: samples analysis and investigation. Shan-Wen Hu: writing-review & editing, supervision, project administration. All authors discussed the results and reviewed the manuscript.

## Conflicts of interest

The authors declare no competing financial interest.

## Supplementary Material

RA-014-D4RA03652B-s001
