# Supplementary material for: Perovskite-based electrochemiluminescence analysis of H2O2
Source: RSC Adv. 2024 Jun 19;14(28):19744–51. doi: 10.1039/d4ra03652b (PMC11188618; doi:10.1039/d4ra03652b)
Supplement: RA-014-D4RA03652B-s001 [file RA-014-D4RA03652B-s001.pdf]

Electronic Supplementary Material (ESI) for RSC Advances.
This journal is © The Royal Society of Chemistry 2024

Perovskite-Based Electrochemiluminescence Analysis of H_2O_2

Ziyi Jia,[#] Hui Zhang, [#] Yuxin Chen, Yuan Fang, Junnan Zhang, Shanwen Hu*

Department of Health Inspection and Quarantine, School of Public Health, Fujian Medical University, Fuzhou, Fujian, 350122, P.R. China.

Table of Contents

Table.S1 Fitting parameters of the PL decay curve

Figure.S1 Excitation and emission spectra of graphene quantum dots.

Figure.S2 DPV curves of CTAB CsPbBr3/GCE under different conditions.

Figure.S3 Optimization of experimental conditions.

Figure.S4 Long time testing of the ECL signal.

Table.S1 Fitting parameters of the PL decay curve

Sample	τ_1 (ns)	τ_2 (ns)	τ_3 (ns)	τ_{ave} (ns)
$CsPbBr_3$	2.03	8.96	71.38	2.03
CTAB-$CsPbBr_3$	4.80	19.87	122.87	4.82

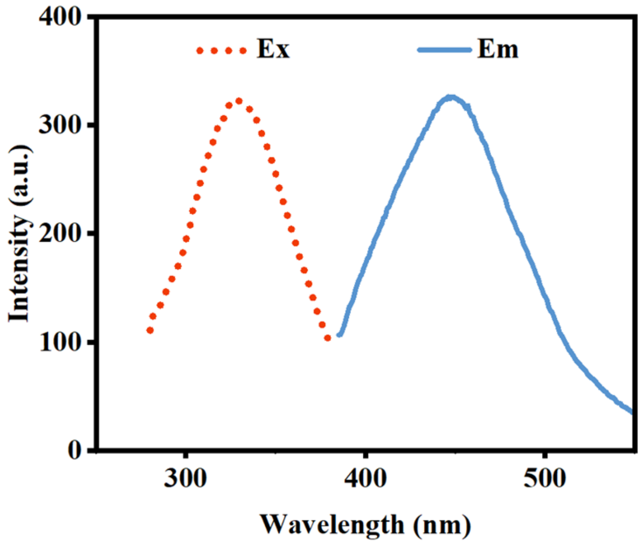

Fig.S1 The excitation (red line) and emission (blue line) spectrogram of graphene quantum dots

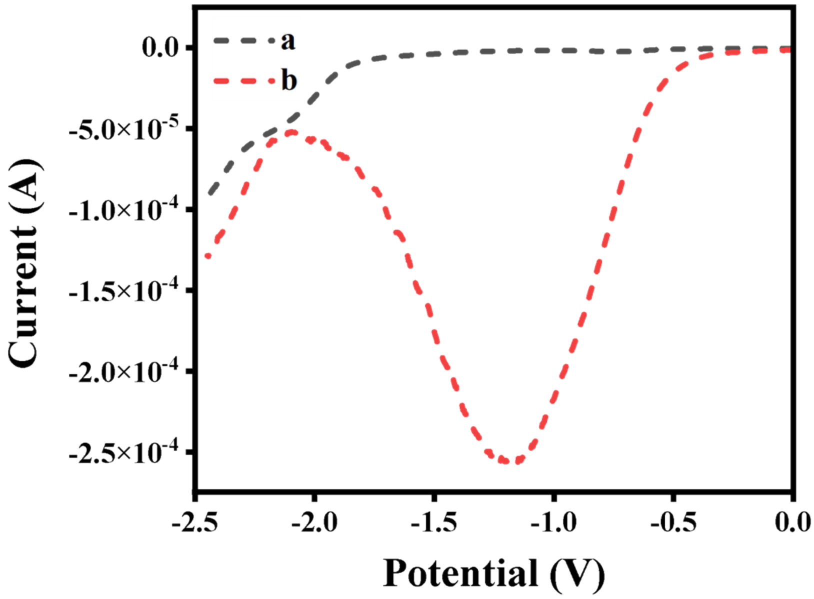

Fig.S2 DPV curves of CTAB $CsPbBr_3$/GCE in 0.1 M PBS solution (curve a) and in PBS solution containing 0.2 M potassium persulfate (curve b)

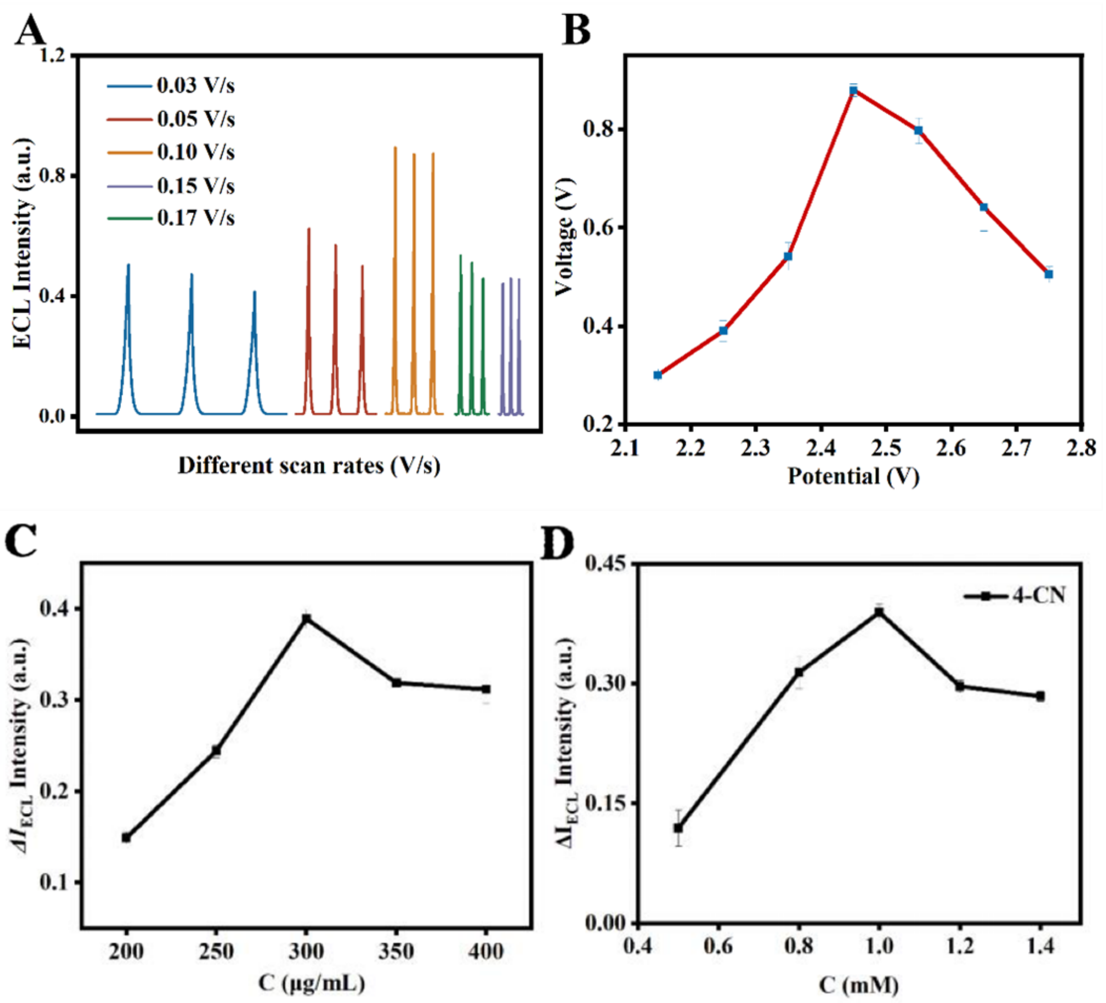

Fig.S3 (A) Optimization of scanning rate for CTAB $CsPbBr_3$/GCE electrochemiluminescence; (B) Optimization of CTAB $CsPbBr_3$/GCE electrochemiluminescence scanning potential range; (C) Optimization of HRP concentration; (D) Optimization of 4-CN concentration

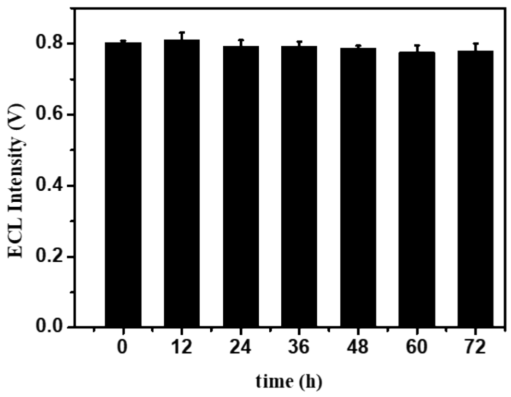

Figure.S4 Long time testing of the ECL signal.
